# Activated Protein C Induces Endoplasmic Reticulum Stress and Attenuates Lipopolysaccharide-Induced Apoptosis Mediated by Glycogen Synthase Kinase-3**β**

**DOI:** 10.1155/2012/485279

**Published:** 2012-05-28

**Authors:** Liang Luo, Tangfeng Lv, Qian Wang, Ting Zhang, Xiaoling Gu, Feng Xu, Yong Song

**Affiliations:** ^1^Department of Respiratory Medicine, Jinling Hospital, Nanjing University School of Medicine, Nanjing 210002, China; ^2^Intensive Care Unit, Wuxi Second Affiliated Hospital, Nanjing Medical University, Wuxi 210004, China; ^3^Department of clinical Laboratory, Wuxi Maternity and Child Health Affiliated Hospital, Nanjing Medical University, Wuxi 210004, China; ^4^Department of Respiratory Medicine, Second Affiliated Hospital, Zhejiang University School of Medicine, Hangzhou 310009, China

## Abstract

This study investigated the relationship between antiapoptotic activities induced by activated protein C and endoplasmic reticulum stress. In this study, it was observed that activated protein C elicited a rise in glucose-regulated protein 78 and glycogen synthase kinase-3**β** and inhibited apoptosis in human umbilical vein endothelial cells induced by lipopolysaccharide. Calcium inhibition did not alter the antiapoptotic effect of activated protein C. The antiapoptotic efficiency of activated protein C was reduced in human umbilical vein endothelial cells following treatment with glycogen synthase kinase-3**β**-siRNA. In summary, activated protein C induced endoplasmic reticulum stress and attenuated lipopolysaccharide-induced human umbilical vein endothelial cell apoptosis mediated by glycogen synthase kinase-3**β**.

## 1. Introduction

Activated protein C (APC), a vitamin-K-dependent serine protease, plays an essential role in blood coagulation together with its cofactor, protein S [1–3]. In recent years, many studies have provided clinical evidence of improved outcomes in severe sepsis following treatment with recombinant APC treatment [4, 5]. It is a pharmacologic success, and international guidelines have recommended the inclusion of recombinant APC in the management of severe sepsis [6, 7]. However, the underlying mechanism this effect remains to be elucidated.

APC exhibits pleiotropic characteristics in addition to its anticoagulant properties of APC [1–3], including anti-inflammatory activities, antiapoptotic activity, and protection of endothelial barrier function [8, 9]. Several studies have shown that the mitochondrion is the main site of the antiapoptotic activity of APC [9]. However, the involvement of APC in the induction of endoplasmic reticulum (ER) stress and inhibition of apoptosis has not been determined. Therefore, this study aimed to investigate the relationship between antiapoptotic activities induced by APC and ER stress in order to elucidate the mechanism of the cytoprotective effects of APC.

ER is the site of synthesis, folding, maturation, and transport of unfolded proteins [10, 11]. ER stress increases unfolded protein load and causes destabilization of cellular calcium [10–13]. To reduce the accumulation of misfolded proteins in the ER, the unfolded protein response (UPR), an adaptive response, promotes and reestablishes ER homeostasis and increases ER folding capacity. Glucose-regulated protein 78 (GRP78), a classic molecular chaperone, interacts with exposed hydrophobic areas of protein-folding intermediates and is responsible for maintaining the protein in a folding-competent state [10–13]. To overcome the destabilization of cellular calcium, calcium flux from the ER to the mitochondria or cytoplasma maintains ER calcium homeostasis [13]. Furthermore, ER stress induces an antiapoptotic effect via a pathway involving glycogen synthase kinase-3*β* (GSK-3*β*) [14].

There is evidence that APC treatment modulates gene expression in endothelial cells (ECs) [9], which increase the workload of the ER. Moreover, APC may induce calcium release from the ER in human brain ECs and human umbilical vein endothelial cells (HUVECs) by binding to endothelial protein C receptor (EPCR) and signaling via protease-activated receptors-1 (PAR-1) [13]. Therefore, it is hypothesized that APC act as an ER stressor and elicit ER stress. However, APC upregulation of GRP78, a marker of ER stress, the impact of calcium release from the ER induced by APC on EC apoptosis, and also APC inhibition of LPS-induced apoptosis mediated by GSK-3*β* are issues that remain to be clarified.

In this study, the effect of APC treatment on GRP78 protein expression was investigated and the relationship with lipopolysaccharide-(LPS-) induced apoptosis was evaluated. The specific inhibitor of calcium release from the ER, 8-(N, N-diethylamino)-octyl-3, 4, 5-trimethoxybenzoate (TMB-8), was used to investigate the effect of calcium release from ER induced by APC on LPS-induced apoptosis. Moreover, GSK-3*β* protein expression following APC treatment was analyzed and GSK-3*β*-siRNA was employed to assess its role in the mechanism underlying the anti-apoptotic effect of APC. It was observed that APC-induced ER stress and attenuated LPS-induced apoptosis mediated by GSK-3*β*, thus suggesting the existence of a novel antiapoptotic mechanism utilized by HUVECs allowing adaptation to LPS challenge.

## 2. Materials and Methods

### 2.1. Reagents and Antibodies

HUVECs were obtained from ScienCell Research Laboratories (San Diego, CA, USA). RPMI 1640 medium, 10% fetal bovine serum (FBS), and Opti-MEM were obtained from GIBCO (Grand Island, NY, USA). APC, LPS, Annexin V-FITC, and propidium iodide (PI) were obtained from Sigma (St. Louis, MO, USA). TMB-8 was obtained from Aldrich Chemical Co. (Milwaukee, WI, USA). The following antibodies were used for Western blots: monoclonal mouse antibodies against human GRP78 (Bioworld Technology, Minneapolis, MN, USA), GSK-3*β* (Santa Cruz Biotechnology, Santa Cruz, CA, USA), and GAPDH (KangChen, Shanghai, China). Protein assay and ECL kits were obtained from Santa Cruz Biotechnology (Santa Cruz, CA, USA). Lipofectamine 2000 (11668-027) was obtained from Invitrogen (Carlsbad, CA, USA). BCA protein assay kit was obtained from Pierce Chemical Co. (Rockford, IL, USA).

### 2.2. Cell Culture

HUVECs were cultured in RPMI 1640 medium supplemented with 10% FBS, 100 *μ*g/mL penicillin, and 100 U/mL streptomycin, at 37°C in a 5% CO_2_—95% air-humidified incubator (Heraeus, Germany). Cells were trypsinized and passaged weekly. The cells were then seeded on 60 mm dishes and treated experimentally after overnight culture.

### 2.3. Small Interference RNA (siRNA) Transfection

Cells were grown overnight in 12-well plates to a density of 3-4 × 10^4^ viable cells per well and transfected with siRNA for GSK-3*β* (Genepharma, Shanghai, China) using Lipofectamine 2000 transfection reagent according to the protocol provided by the manufacturer. After 24 h, media was exchanged and the cells were treated with LPS for an additional 24 h. Transfection efficiency of GSK-3*β*-siRNA was assessed by RT-PCR for GSK-3*β* mRNA and immunoblotting for GSK-3*β* protein. APC treatment (150 nM) was conducted for 0, 6, 12, and 24 h. Finally, cells were harvested for apoptosis assays and Western blot analysis.

### 2.4. Apoptosis Assays

Approximately 2 × 10^5^ cells/well of HUVECs in 12-well plates at concentrations of 10 ng/mL LPS were incubated in an incubator for 24 hours, and subsequently treated with 150 nM APC for 0, 6, 12, and 24 h. Cells were trypsinized and washed with PBS, then harvested by centrifugation. The cells were resuspended in PBS, followed by PI-Annexin V-FITC staining. Flow cytometric analysis of PI-Annexin V-FITC staining was conducted according to the instructions provided by the manufacturer for quantification of apoptosis. Results represent the mean of triplicate determinations in which a minimum of 10,000 cells were assayed for each determination.

### 2.5. Western Blot Analysis

Cells were grown in 100 mm dishes to a density of 5–7 × 10^5^ viable cells per dish, and then were pretreated with 10 ng/mL LPS for 24 h before the addition of 150 nM APC for 0, 6, 12, and 24 h. Subsequently, cells were trypsinized and washed with PBS, then harvested by centrifugation for following experiments. At varying time points during APC treatment, cells were lysed in Triton lysis buffer (20 mM Tris, pH 7.4, 137 mM NaCl, 10% glycerol, 1% Triton X-100, 2 mM EDTA, 1 mM PMSF, 10 mM NaF, 5 mg/mL aprotinin, 20 mM leupeptin, and 1 mM sodium orthovanadate) and centrifuged at 12,000 g for 15 min. Protein concentrations were measured using the BCA assay. Protein samples were resolved by SDS polyacrylamide gel electrophoresis (SDS-PAGE), transferred to polyvinylidene fluoride (PVDF) membranes, and then blocked with 5% skimmed milk powder containing 0.1% Tween-20. Blots were then probed at 4°C overnight with relevant antibodies, washed with TBST (TBS containing 0.1% Tween-20) three times, and probed with the appropriate horseradish peroxidase-conjugated secondary antibodies at room temperature for 2 h. The relative abundance of each protein was determined by scanning densitometry using GAPDH as an internal control. All immunoblots were visualized by ECL.

### 2.6. Statistical Analysis

At least three independent experiments were conducted for each treatment. Comparisons within groups were made using an appropriate Student's* t*-test. *P* < 0.05 was considered statistically significant.

## 3. Results

### 3.1. APC Attenuation of LPS-Induced Apoptosis in HUVECs

**Figure 1:**
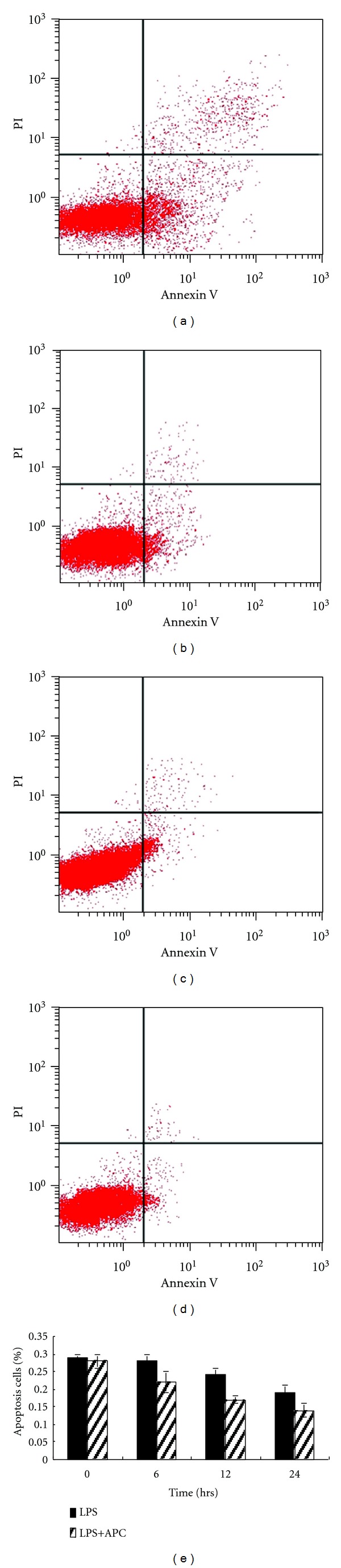
APC attenuated LPS-induced apoptosis in HUVECs. Flow cytometric analysis of cultured HUVECs continuously exposed to 10 ng/mL LPS for 24 h revealed a high percentage of apoptotic cells (a). Subsequently, LPS-stimulated HUVECs were treated with 150 nM APC for 0, 6, 12 and 24 h ((a), (b), (c), and (d)). APC inhibition of cell apoptosis was detected following 6 h exposure ((b), (e); *P* < 0.05) and then generally decreased. Flow cytometric analysis of PI-Annexin V-FITC staining revealed that 150 nM APC treatment markedly attenuated LPS-induced apoptosis in HUVECs.

As an initial step to clarify the antiapoptotic mechanism of APC, the effect of APC treatment on HUVECs survival following LPS stimulation was examined. Cells were continuously exposed to 10 ng/mL LPS for 24 h and subsequently treated with 150 nM APC for 0, 6, 12 and 24 h. It was observed that APC inhibited cell apoptosis markedly after 6 h of APC treatment. Flow cytometric analysis of PI-Annexin V-FITC staining revealed that APC treatment markedly decreased the number of apoptotic cells (*P* < 0.05). Collectively, these data show that 150 nM APC treatment attenuated LPS-induced apoptosis in HUVECs (Figure 1).

### 3.2. APC Induction of UPR in the ER

**Figure 2:**
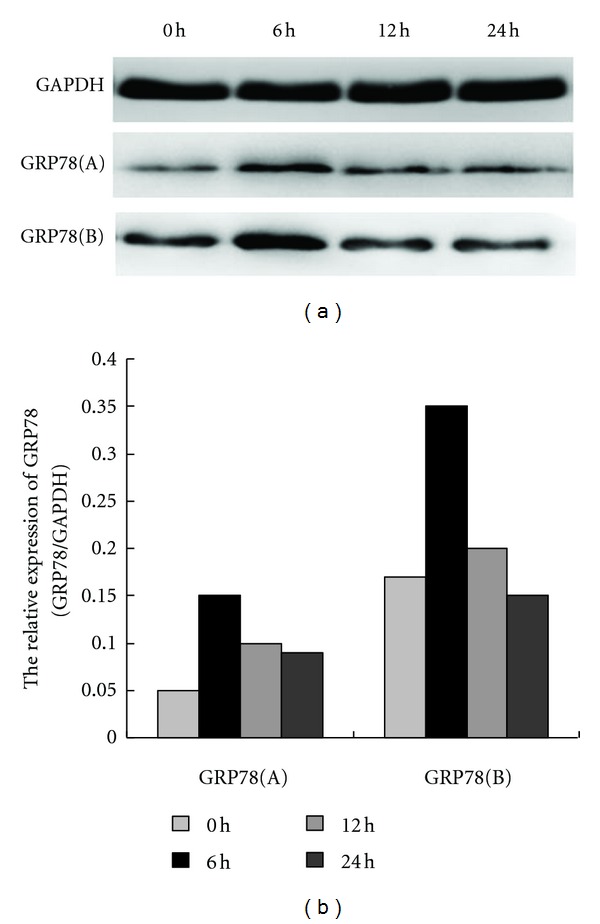
APC-induced GRP78 expression in the presence and absence of LPS stimulation. Following HUVEC treatment with 150 nM APC for 0, 6, 12, and 24 h, upregulated GRP78 expression was initially detected at 6 h by Western blot analysis (GRP78 (a), *P* < 0.05), and then generally decreased. After continuous exposure to 10 ng/mL LPS for 24 h, HUVECs were treated with 150 nM APC for 0, 6, 12, and 24 h. Upregulated GRP78 expression was also detected at 6 h by Western blot analysis (GRP78 (b), *P* < 0.05), and then generally decreased. A significant difference in APC-induced GRP78 expression in the presence (b) and absence (a) of LPS stimulation was detected at 6 h, which indicated that 150 nM APC plays a more significant role in this effect following LPS stimulation (*P* < 0.05).

Evidence has been obtained in previous studies that 150 nM APC induces a transient rise in calcium release from ER in HUVECs, implying an ER disturbance evoked by APC [13]. To investigate ER stress induced by APC, the effect of an ER-stress-related protein, GRP78 on HUVECs was examined following exposure for 0, 6, 12, 24 h. APC treatment upregulated GRP78 protein expression at 6 h (Figure 2). Upregulated GRP78 is responsible for the UPR in the ER. Therefore, these data indicate that APC acts as an ER stressor and elicits the UPR.

### 3.3. Calcium Inhibitor Did Not Alter the Antiapoptotic Effect of APC

**Figure 3:**
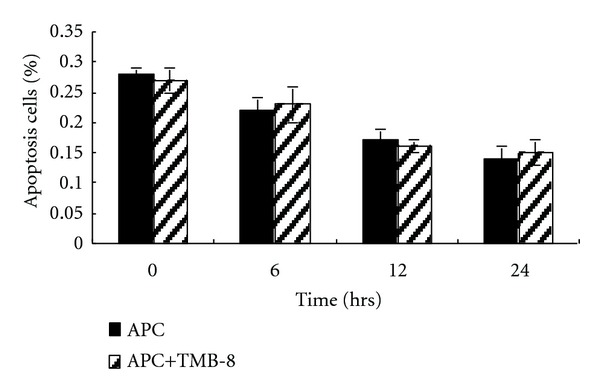
Calcium inhibition did not alter the antiapoptotic effect of APC. After exposure to 10 ng/mL LPS for 24 h, HUVECs were treated with 150 nM APC for 0, 6, 12, and 24 h in the presence or absence of 50 *μ*M TMB-8, an inhibitor of calcium release from the ER. There was no significant difference in the percentage of apoptosis cells between the two groups, which was detected by flow cytometric analysis (APC versus APC + TMB-8, *P* > 0.05).

The inhibitor of calcium release, TMB-8, was used to investigate the influence of the transient rise of intracellular calcium-induced by APC on HUVEC apoptosis. The effective TMB-8 dose for inhibition of calcium release from the ER has been verified to be 50 *μ*M. Therefore LPS-pretreated HUVECs were exposed to 150 nM APC in the presence or absence of TMB-8 (50 *μ*M) for 0, 6, 12, and 24 h. Apoptosis of HUVECs were examined by flow cytometric analysis. No significant changes in apoptosis was observed in LPS-pretreated HUVECs between the two groups (APC versus APC + TMB-8, *P* > 0.05) (Figure 3).

### 3.4. Upregulation of GSK-3*β* by APC and Reduction in Antiapoptotic Activity Following GSK-3*β*-siRNA Treatment

**Figure 4:**
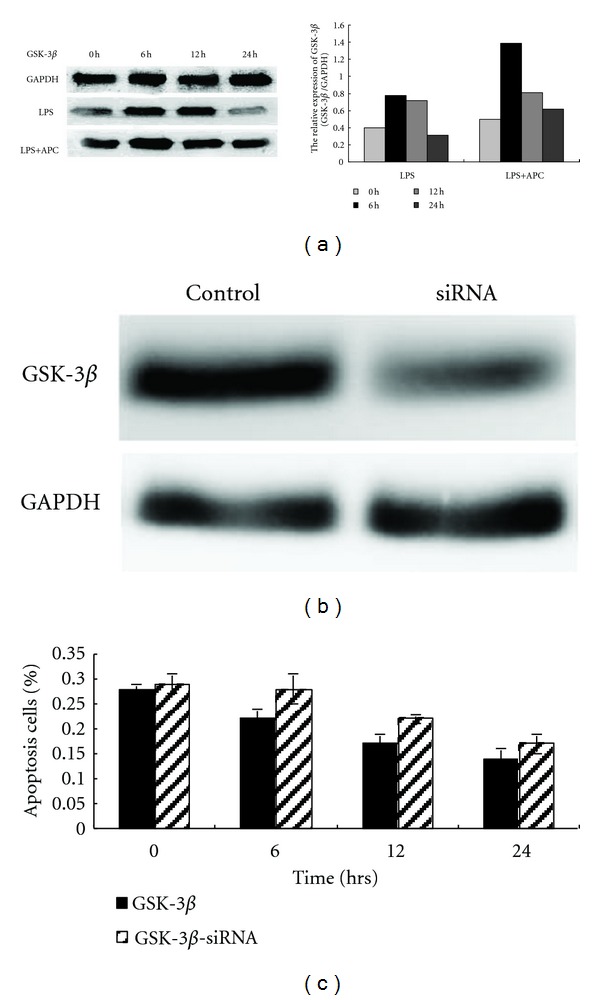
Upregulation of GSK-3*β* expression following APC treatment and decreased antiapoptotic activity following GSK-3*β*-siRNA transfection of HUVECs. HUVECs exposed to 10 ng/mL LPS for 24 h, were treated with 150 nM APC for 0, 6, 12, and 24 h. Upregulated GSK-3*β* expression was detected, peaking at 6 h after APC treatment (a). Transfection of HUVECs with GSK-3*β*-siRNA decreased the abundance of GSK-3*β* protein significantly (b). Compared with normal HUVECs treated with 150 nM APC, a significant increase of the percentage of apoptosis cells was found in HUVECs pretreated with GSK-3*β*-siRNA (GSK-3*β* versus GSK-3*β*-siRNA, *P* < 0.05), indicating that the antiapoptotic activity of APC was inhibited by GSK-3*β*-siRNA (c).

APC treatment elicited a rise in GSK-3*β* expression, peaking after 6 h APC treatment. The level of GSK-3*β* generally decreased with prolonged APC treatment (Figure 4(a)). To further substantiate the role of GSK-3*β*, GSK-3*β*-siRNA was used to inhibit the expression of GSK-3*β*. HUVECs transfected by GSK-3*β*-siRNA showed a substantial reduction in GSK-3*β* mRNA compared to the control by RT-PCR analysis (GSK-3*β*-siRNA versus control, *P* < 0.05). Transfection of HUVEC with GSK-3*β*-siRNA decreased the abundance of GSK-3*β* protein significantly (Figure 4(b)). Compared with normal HUVECs treated with 150 nM APC, a significant increase in the number apoptotic cells was detected in HUVECs pretreated with GSK-3*β*-siRNA (Figure 4(c)), thus indicating that antiapoptotic activity of APC was partially inhibited by GSK-3*β*-siRNA.

## 4. Discussion

This study demonstrated that APC induces ER stress and elicited the GSK-3*β*-mediated antiapoptotic effect, thus attenuating LPS-induced apoptosis. Further evidence of the role of APC as an ER stressor was also obtained. A series of intrinsic and extrinsic ER stressors have been reported, such as hypoxia, ischemic, and reperfusion injury, nutrient deprivation, pH change, calcium depletion from the ER lumen, inhibition of asparagine (N)-linked glycosylation, reduction of disulfide bonds, and overexpression of some proteins [10–12]. These ER stressors cause two types of characteristic changes in the ER: upregulation of molecular chaperones such as GRP78 and calcium release [13]. Dömötör et al. reported that HUVECs challenged with 150 nM APC responded with a transient elevation in intracellular calcium. After ER calcium stores were depleted using thapsigargin (TG), subsequent exposure of cells to APC did not evoke an intracellular calcium signal, confirming the ER as the origin of APC-evoked calcium signals [13]. In this study, it was demonstrated that treatment of cultured HUVECs with 150 nM APC elicited a rise in GRP78 expression in parallel with the attenuation of apoptosis in HUVECs over time (Figure 1). While GRP78 was upregulated at 6 h, the percentage of apoptotic HUVECs was markedly reduced at the same time. While the level of GRP78 declined at 12 h and 24 h, the percentage of apoptotic HUVECs was markedly reduced at the same time. These observations confirmed that APC acts as an ER stressor and induces a UPR in the ER, which in turn, plays an antiapoptotic role in LPS-stimulated HUVECs. 

ER is the main intracellular storage compartment for calcium. The close physical contact of mitochondria and the ER results in the mitochondria being exposed to more intracellular calcium than the rest of cytosol when intracellular calcium is released from the ER. Calcium overload of mitochondria promotes permeabilization of the outer mitochondrial membrane and the release of several apoptogenic factors resulting in cell apoptosis [15, 16]. However, Deniaud reported that ER stress agents and physiological stimuli generated calcium flux of the mitochondrial compartment, but cell apoptosis or death was indeterminate. Histamine is a typical ER stressor that induces a transient calcium increase without cell death [17]. However, it is not known whether the transient intracellular calcium elevation evoked by APC causes calcium-induced mitochondrial apoptosis. In this study, TMB-8, a specific inhibitor of calcium release from the ER, was used to block calcium transfer from the ER to the mitochondrion. As a result, LPS-induced apoptosis was not attenuated, which implied that APC-evoked intracellular calcium elevation did not cause calcium-induced mitochondrial apoptosis.

GSK-3*β* was initially described as a key component of the glycogen metabolic pathway. GSK-3*β* is also a multifunctional serine/threonine kinase, involved in the phosphorylation of transcription and translation factors as well as regulation of the cell cycle and proliferation [18, 19]. ER stress induces p53 phosphorylation by GSK-3*β*, which facilitates nucleocytoplasmic export and degradation of p53 [14]. In this study, APC treatment was shown to elicit upregulation of GSK-3*β* in normal HUVECs. In GSK-3*β*-siRNA-treated HUVECs, the expression of GSK-3*β* was inhibited, with a subsequent decrease in the antiapoptotic efficiency of APC, thus indicating that GSK-3*β* is an essential mediator of the antiapoptotic effect of APC.

In summary, this study demonstrated that APC-induced ER stress in HUVECs and attenuated LPS-induced HUVEC apoptosis mediated by GSK-3*β*.

## References

[1] Katsuura Y, Mochizuki T, Tamura M (1996). Species specificity of anticoagulant activity of activated human protein C: involvement of factor V as well as protein S. *Thrombosis Research*.

[2] Riewald M, Ruf W (2003). Science review: role of coagulation protease cascades in sepsis. *Critical Care*.

[3] Lundy DJ, Trzeciak S (2009). Microcirculatory dysfunction in sepsis. *Critical Care Clinics*.

[4] Ferrer R, Artigas A, Suarez D (2009). Effectiveness of treatments for severe sepsis: a prospective, multicenter, observational study. *American Journal of Respiratory and Critical Care Medicine*.

[5] Hodder RV, Hall R, Russell JA, Fisher HN, Lee B (2009). Early drotrecogin alpha (activated) administration in severe sepsis is associated with lower mortality: a retrospective analysis of the Canadian ENHANCE cohort. *Critical Care*.

[6] Martin G, Brunkhorst FM, Janes JM (2009). The international PROGRESS registry of patients with severe sepsis: drotrecogin alfa (activated) use and patient outcomes. *Critical Care*.

[7] Dellinger RP, Levy MM, Carlet JM (2008). Surviving Sepsis Campaign: international guidelines for management of severe sepsis and septic shock: 2008. *Critical Care Medicine*.

[8] Faffe DS, Seidl VR, Chagas PSC (2000). Respiratory effects of lipopolysaccharide-induced inflammatory lung injury in mice. *European Respiratory Journal*.

[9] Mosnier LO, Zlokovic BV, Griffin JH (2007). The cytoprotective protein C pathway. *Blood*.

[10] Faitova J, Krekac D, Hrstka R, Vojtesek B (2006). Endoplasmic reticulum stress and apoptosis. *Cellular and Molecular Biology Letters*.

[11] Boyce M, Yuan J (2006). Cellular response to endoplasmic reticulum stress: a matter of life or death. *Cell Death and Differentiation*.

[12] Rasheva VI, Domingos PM (2009). Cellular responses to endoplasmic reticulum stress and apoptosis. *Apoptosis*.

[13] Dömötör E, Benzakour O, Griffin JH, Yule D, Fukudome K, Zlokovic BV (2003). Activated protein C alters cytosolic calcium flux in human brain endothelium via binding to endothelial protein C receptor and activation of protease activated receptor-1. *Blood*.

[14] Qu L, Huang S, Baltzis D (2004). Endoplasmic reticulum stress induces p53 cytoplasmic localization and prevents p53-dependent apoptosis by a pathway involving glycogen synthase kinase-3*β*. *Genes and Development*.

[15] Deniaud A, Sharaf El Dein O, Maillier E (2008). Endoplasmic reticulum stress induces calcium-dependent permeability transition, mitochondrial outer membrane permeabilization and apoptosis. *Oncogene*.

[16] Zhang D, Lu C, Whiteman M, Chance B, Armstrong JS (2008). The mitochondrial permeability transition regulates cytochrome c release for apoptosis during endoplasmic reticulum stress by remodeling the cristae junction. *Journal of Biological Chemistry*.

[17] Mosnier LO, Griffin JH (2003). Inhibition of staurosporine-induced apoptosis of endothelial cells by activated protein C requires protease-activated receptor-1 and endothelial cell protein C receptor. *Biochemical Journal*.

[18] Doble BW, Woodgett JR (2003). GSK-3: tricks of the trade for a multi-tasking kinase. *Journal of Cell Science*.

[19] Cohen P, Frame S (2001). The renaissance of GSK3. *Nature Reviews Molecular Cell Biology*.

